# Takotsubo Cardiomyopathy With Inconspicuous Initial Electrocardiogram: A Potentially Serious Cardiac Pathology Related to Emotional Stress

**DOI:** 10.3389/fpsyt.2019.00308

**Published:** 2019-05-16

**Authors:** Mohamed Elsayed, Bernhard J. Connemann, Tillman Dahme, Temsgen Tesfay, Maximilian Gahr

**Affiliations:** ^1^Department of Psychiatry and Psychotherapy III, University of Ulm, Ulm, Germany; ^2^Department of Medicine II, Ulm University Medical Center, Ulm, Germany

**Keywords:** acute coronary syndrome, major depressive disorder, Takotsubo cardiomyopathy, electrocardiagram, emotional stress

## Abstract

**Introduction:** Takotsubo cardiomyopathy (TCM) is frequently associated with emotional or physical stress. Thus, patients with TCM might present primarily at a psychiatric clinic. Appropriate diagnosis and therapy may thus be delayed.

**Case report:** A 43-year-old female patient presented as an emergency to the psychiatric outpatient clinic after experiencing severe work-related bullying. On admission, she complained of acute left thoracic chest pain as well as depressed mood, low energy, anhedonia, generalized anxiety, and sleep difficulties, present for several weeks. The initial electrocardiogram (ECG) was unremarkable; serum troponin levels, however, were markedly elevated. The patient was transferred to the department of cardiology. *Via* cardiac catheterization and MRI, an acute coronary syndrome was excluded and apical ballooning and left ventricular dysfunction, compatible with TCM, was found.

**Conclusion:** Patients with acute psychopathology, recent emotional or physical stress, and acute cardiothoracic symptoms should receive immediate cardiological investigations. As the ECG may be normal in patients with TCM, concurrent measurement of the troponin serum level is recommended. Psychiatrists should consider TCM in patients who report recent stressful events accompanied by cardiothoracic symptoms.

## Introduction

Takotsubo cardiomyopathy (TCM) is an acute and transient left ventricular wall-motion abnormality that is frequently associated with emotional or physical stress (1). Symptoms of TCM may mimic myocardial infarction (2) and include chest pain, dyspnea, and hypotension. The electrocardiogram (ECG) might show ST-segment changes or T-wave inversions (3). Typically, troponin is elevated (4). Diagnostic investigations include electrocardiography, coronary angiography, and cardiac MRI (3) (for diagnostic criteria, see **Table 1**). Treatment involves monitoring and treatment of potential complications (5). Hospital mortality rates are approximately 2% (6). The etiology of TCM is not well understood. Current pathophysiologic hypotheses consider the hypothalamic–pituitary–adrenal axis and sudden catecholamine excess (4, 7). In about two-thirds, TCM is preceded by significant emotional stress (5). Therefore, patients with TCM might present at first at a psychiatric clinic, which may impede adequate diagnosis and therapy. In this regard, we present and discuss such a patient in order to increase awareness regarding TCM.

**Table 1:** Modified Mayo Clinic Criteria for Takotsubo cardiomyopathy (3).

1. Transient hypokinesia or akinesia of left ventricle with regional wall motion abnormality, majority involving apex and mid left ventricle (or other areas) extending beyond the distribution of single epicardial artery; hypokinesia invariably (but not always) follows stressful trigger, which could be emotional or physical.
2. Appearance of new ECG abnormalities like ST elevation, T inversion, Q waves with mild elevation of troponins and pro-brain natriuretic peptide (pro-BNP) markers.
3. Absence of obstructive lesion (plaque rupture, thrombus, or spasm) of epicardial coronary artery [thus excluding ST-elevation myocardial infarction (STEMI), Non-ST-elevation myocardial infarction (NSTEMI), and Prinzmetal angina].
4. Absence of phaeochromocytoma and myocarditis.

## Case Presentation

A 43-year-old woman presented in 2018 as an emergency to our psychiatric outpatient department. She reported depressed mood, sleep difficulties, and loss of energy, present for several weeks. Two hours earlier, she had been severely verbally offended by her colleague, which had induced emotional stress and led her to introduce herself to our clinic. Psychopathological findings on time of admission were anxiety, depressed mood, anger, loss of drive, anhedonia, and insomnia, consistent with major depressive disorder. Use of psychotropic substances was denied. The family history was positive for depressive disorder. She was a smoker. The somatic history revealed hypertension and neurodermatitis. The daily medication was diclofenac 75 mg once daily (OD). On admission, she additionally complained of persistent, non-respiration-dependent left-thoracic chest pain, lasting for about 2 h prior to admission; in addition, hyperventilation, symmetric leg tingling, and heaviness had started together with the chest pain; however, it had resolved about 30 min prior to admission. No past episodes of dyspnea or disturbances of consciousness were reported. Physical examination and resting ECG, performed immediately after introduction to our outpatient department, were unremarkable. Laboratory investigations revealed a significantly elevated troponin T (243 ng/ml; reference <14 ng/ml). We then transferred the patient to the department of cardiology, where another unremarkable ECG was performed (findings: sinus rhythm, heart rate 84/min, normal cardiac axis, the transition from S>R wave to R>S wave was between V3 and V3, no repolarization disorders). An emergency cardiac catheterization, which also included coronary angiography and ventriculography, was performed on the day of presentation in the department of cardiology and demonstrated severely reduced left ventricular function with typical apical ballooning; a coronary heart disease was excluded. The N-terminal (NT)-pro-brain natriuretic peptide (BNP) levels were elevated at 307.0 pg/ml (reference: <130.0 pg/ml). A cardiac MRI was performed about 47 h after the initial presentation in the psychiatry outpatient department and showed a nonhypertrophied left ventricle with mildly reduced systolic function with an ejection fraction of 52%, consistent with TCM; in the cardiac MRI, the right ventricle was not hypertrophied and its ejection fraction was 55%; there was evidence of a beginning diastolic dysfunction in the left and right ventricles and no evidence of myocardial scars or fibrosis. The patient was monitored for 5 days in the department of cardiology and was then discharged without any cardiac symptoms or complications; on follow-up 8 weeks later, the clinical investigation was unremarkable.

## Discussion

In the present case, the occurrence of TCM was preceded by depressive symptoms related to major depressive disorder for several weeks; in addition, acute social stress (work-related bullying) had occurred immediately before. Regarding the pathophysiology of TCM, previous studies suggest that mental stress causes disruption of endothelial function through endothelin-A receptors even in healthy individuals (8, 9), which may also have been relevant in our case. Moreover, Nayeri et al. performed a retrospective study and observed that a preexisting psychiatric illness was associated with an increased risk of recurrent TCM (10), making exacerbation of psychiatric disorders a possible risk factor for TCM. Typical features of TCM are female gender and emotional stress, which precedes the onset of cardiothoracic symptoms (11, 12). These features were also present in our case; the occurrence of work-related bullying in connection with a preexisting major depressive disorder represents a serious stressful event. In this respect, our case is a typical case of TCM. By contrast, considering that about 85% of TCM patients feature more or less unspecific ECG changes (13), the complete absence of ECG abnormalities—as found in our case—is a rather rare finding in TCM. Furthermore, the age of onset of TCM in this patient was almost 25 years earlier than the mean age of onset found in the study by Templin et al. (14). In this regard, it should be considered that the typical apical ballooning in our case is rare in premenopausal women, who often present atypical variants of TCM such as akinesia of the mid-basal portion and hyperkinesia of the apical segments (15). Despite unremarkable ECG findings and a comparatively low age, the patient had elevated troponin serum levels and the results of the cardiac MRI were typical of TCM.

TCM, however, may be precipitated not only by emotional stress (16, 17). Also physical stress, e.g., neurovascular events, may precipitate TCM: transient left ventricular dysfunction frequently occurs in association with subarachnoid hemorrhage (16, 17), and thus clinical and pathophysiological similarities with TCM are discussed in the literature (16, 17). Regarding the management of patients with acute cerebrovascular events or head trauma, who regularly feature disturbances of consciousness and thus cannot contribute relevant anamnestic data (e.g., cardiothoracic symptoms), it is important to consider the possibility of neurogenic cardiomyopathies and to perform adequate cardiologic diagnostic procedures (echocardiography, cardiac catheterization with coronary angiography, and ventriculography) (17). Our case did not feature head trauma or a cerebrovascular event; however, transient severely reduced left ventricular function subsequent to severe emotional stress was found, supporting the concept of neurogenic cardiomyopathies that can be induced by mental or physical stress (16, 17).

Apart from psychiatric disorders, some psychiatric treatment modalities were reported to be associated with TCM: cases of serotonin norepinephrine reuptake inhibitor-induced TCM through excess catecholamine secretion have been reported (18). Treatment with lithium potentiates secretion of catecholamines and could therefore induce TCM (19). In addition, electroconvulsive therapy might also trigger TCM (20).

## Conclusion

Our case supports the concept that TCM can be triggered by emotional stressors and that comorbid mental disorders might increase the risk of TCM. Patients with acute psychopathology, recent emotional or physical stress, and cardiothoracic symptoms should receive immediate cardiological investigations. As the ECG may be normal in patients with TCM, concurrent measurement of the troponin serum level is recommended. Psychiatrists should consider TCM in patients who report recent stressful events accompanied by cardiothoracic symptoms. As the presence of a mood disorder appears to be a possible risk factor for TCM, patients with mood disorders should undergo screening by ECG and troponin serum levels and, if pathological, immediately referred to a cardiologist.

## Data Availability Statement

All datasets generated for this study are included in the manuscript and/or the supplementary files.

## Ethics Statement

Written informed consent was obtained from the patient regarding the use of her anonymous data for scientific and research reasons and for publication of this case report and any accompanying images.

## Author Contributions

ME prepared the manuscript, TD and TT helped in the implementation. BC supervised the manuscript. MG conceived in the manuscript and participated in its design and coordination and helped to draft the manuscript. All authors read and approved the final manuscript.

## Conflict of Interest Statement

The authors declare that the research was conducted in the absence of any commercial or financial relationships that could be construed as a potential conflict of interest.
